# Human oocyte developmental potential is predicted by mechanical properties within hours after fertilization

**DOI:** 10.1038/ncomms10809

**Published:** 2016-02-24

**Authors:** Livia Z. Yanez, Jinnuo Han, Barry B. Behr, Renee A. Reijo Pera, David B. Camarillo

**Affiliations:** 1 Department of Bioengineering, Stanford University School of Engineering, Stanford, California 94305, USA; 2 Institute for Stem Cell Biology and Regenerative Medicine, Stanford University School of Medicine, Stanford, California 94305, USA; 3 Department of Obstetrics and Gynecology, Stanford University School of Medicine, Stanford, California 94305, USA; 4 Department of Cell Biology, Neuroscience and Chemistry and Biochemistry, Montana State University, Bozeman, Montana 59717, USA

## Abstract

The causes of embryonic arrest during pre-implantation development are poorly understood. Attempts to correlate patterns of oocyte gene expression with successful embryo development have been hampered by the lack of reliable and nondestructive predictors of viability at such an early stage. Here we report that zygote viscoelastic properties can predict blastocyst formation in humans and mice within hours after fertilization, with >90% precision, 95% specificity and 75% sensitivity. We demonstrate that there are significant differences between the transcriptomes of viable and non-viable zygotes, especially in expression of genes important for oocyte maturation. In addition, we show that low-quality oocytes may undergo insufficient cortical granule release and zona-hardening, causing altered mechanics after fertilization. Our results suggest that embryo potential is largely determined by the quality and maturation of the oocyte before fertilization, and can be predicted through a minimally invasive mechanical measurement at the zygote stage.

## Abstract (web summary)

Reliable assessments of oocyte developmental potential are lacking, making it difficult to select the best quality embryos for transfer after *in vitro* fertilization. Here, the authors show that a non-invasive measurement of viscoelastic properties predicts developmental potential in both humans and mice.

## Graphical abstract

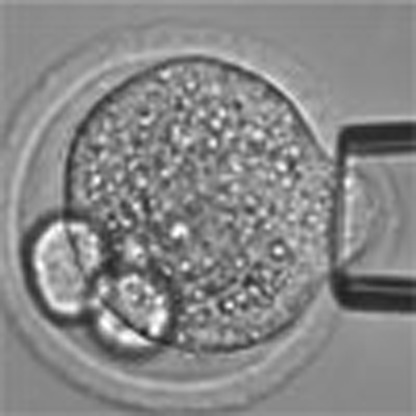

It has been suggested that an embryo's fate is determined very early in development, before embryonic genome activation (EGA), or even before fertilization^1^,^2^,^3^. Before EGA no transcription occurs, and so the very first steps of embryogenesis are controlled exclusively by maternal information inherited from the oocyte^1^,^2^,^3^. The major wave of transcription involved in EGA is observed at the one-cell zygote to the two-cell stage in mouse^4^ and the four- to eight-cell stage in humans^5^. In humans, various oocyte morphological characteristics^6^ have been correlated with embryo development and implantation potential, including zona thickness^7^, granularity^8^, perivitelline space^9^ and oocyte shape^10^. However, these criteria are highly subjective, and their predictive value is controversial^11^. Without a reliable predictor of embryo viability before EGA, it is still unclear to what extent developmental potential is determined by the oocyte.

The mechanisms of developmental failure in embryos resulting from poor-quality oocytes are also largely unknown. Some studies have begun to explore differences at the level of transcription between high- and poor-quality oocytes by inferring quality from maternal age^12^,^13^ or ploidy^14^. However, these measures of oocyte quality can only identify a subpopulation of all non-viable oocytes because chromosomal abnormalities can arise after this stage, and maternal age is not a perfect predictor of viability. An accurate and nondestructive predictor of viability before EGA would allow us a global view of deficiencies in the oocyte transcriptome, which can lead an embryo to arrest.

In recent years, studies have shown that mechanical inputs play a major role in regulating cell fate and function at the molecular level^15^, and a cell's internal state may also be reflected in its mechanical properties^16^,^17^. In mouse and human embryos, cortical granule release during fertilization causes a physical change in stiffness, which is called ‘zona-hardening'^18^, a process previously described only biochemically. The response of the oocyte membrane to needle puncture during intracytoplasmic sperm injection (ICSI) was found to be predictive of embryo morphology and survival in culture^19^,^20^. Embryo and oocyte stiffnesses have also been correlated to pregnancy in humans, and to maternal age in mouse, indicating that there may be a link between mechanics and viability^21^,^22^,^23^.

In this study, we report a set of mechanical parameters that can be measured nondestructively within hours after fertilization and can identify human zygotes destined to arrest, with >90% precision, 95% specificity and 75% sensitivity (Fig. 1). Using these parameters to stratify embryos by viability, we identify important genes and pathways that play key roles in pre-implantation embryo development, most of which are reflective of oocyte nuclear and cytoplasmic maturation, DNA repair and cellular stress response. We also show that patterns of gene expression found in non-viable embryos may affect cortical granule release and zona-hardening, which helps to explain the link between embryo viability and mechanical properties.

## Results

### Zygote mechanical parameters predict blastocyst formation

**Figure 2:**
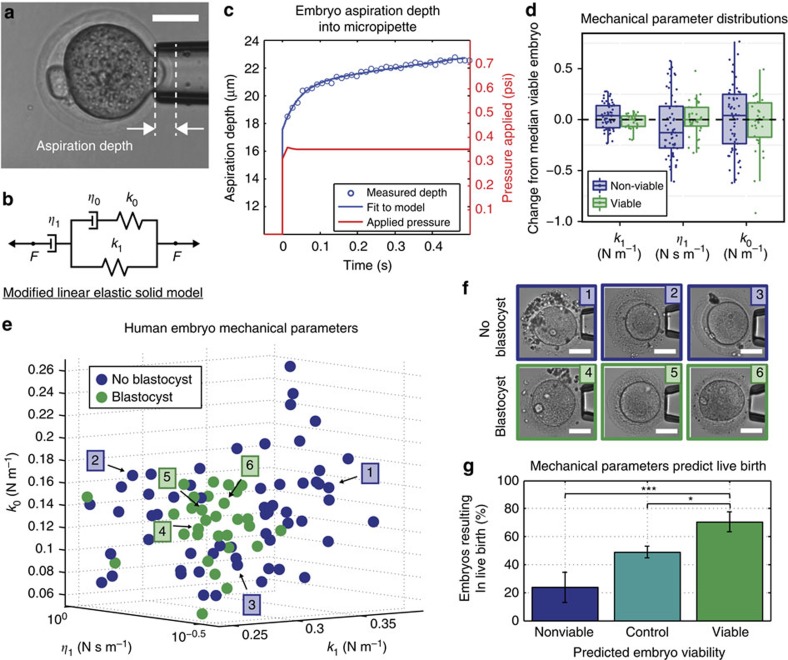
Design of the measurement system and differences in the mechanical parameters between viable and non-viable embryos. (**a**) The measurement technique and (**b**) the mechanical model used to extract mechanical parameters. (**c**) A typical trace of the pressure applied to the embryo through the micropipette (red line) and the response of the embryo to the pressure as it is aspirated into the micropipette (blue line). (**d**) Box-and-whisker plots (box shows median, edges are at first and third quartiles, so total box height is interquartile range (IQR), lower whisker extends from lower edge to lowest value within 1.5*IQR of the edge, upper whisker extends from upper edge to highest value within 1.5*IQR of the edge), with scatterplots overlaid of the mechanical parameters of human zygotes (*n*=89). Parameters of the viable zygotes (*n*=31) appear more tightly clustered than those of non-viable zygotes (*n*=58). (**e**) Three-dimensional scatter plot of three mechanical parameters showing that viable human zygotes (*n*=31) cluster tightly in one region, with non-viable zygotes (*n*=58) scattered around them. (**f**) Example images of some of the human zygotes with similar morphological scores that are predicted to be viable or non-viable based on mechanics. (**g**) The mechanical parameters were also predictive of live birth in mouse, and enabled us to improve live birth rates (*P*=0.01, *χ*^2^-test) compared with a group of control embryos (*n*=55 in each group, over a total of four replicates). *P<0.05, ***P<0.001. Error bars represent s.d. Scale bar, 60 μm.

A total of 89 two-pronuclear (2PN)-stage human zygotes from successful *in vitro* fertilization (IVF) cycles were thawed. We measured their mechanical parameters within 3 h after thawing, and tracked their development over 5–6 days using a time-lapse microscope (Eeva System from Auxogyn), with images taken every 5 min. Those forming blastocysts were designated as viable. Micropipette aspiration was chosen to probe embryo mechanical properties because it is minimally invasive, can be performed quickly and has been extensively used to study the viscoelastic properties of cells^24^,^25^. An image of a mouse zygote undergoing aspiration is shown in Fig. 2a. The four-parameter bulk mechanical model we used to model the embryo is shown in Fig. 2b; it is a Zener^26^ model with an extra viscous element added in series. A sample plot of the pressure applied into the pipette (step function) along with the aspiration depth of the embryo over time and its fit to the mechanical model is shown in Fig. 2c.

Figure 2d shows box plots of three of the four parameters (in order of usefulness in separating non-viable from viable embryos: *k*_1_, *η*_1_, *k*_0_, with *η*_1_ log-transformed). The interquartile range for viable embryo parameters is smaller than that of non-viable embryos, except for the fourth parameter (*η*_0_, Supplementary Fig. 1). A scatter plot of the three parameters from Fig. 2d is shown in Fig. 2e. While the individual parameters in Fig. 2d provide limited predictive value on their own, when plotted together the majority of viable embryos cluster in one region of the scatter plot (*k*_1_=0.30 N m^−1^, *η*_1_=0.59 N s m^−1^ and *k*_0_=0.12 N m^−1^), and there is no ‘typical' non-viable embryo. Figure 2f contains typical images of embryos from Fig. 2e and shows that viable and non-viable embryos are morphologically nearly indistinguishable at this stage in development, but have distinct mechanical parameters. Using the data from Fig. 2e, we trained a support-vector machine (SVM) classifier to determine the optimal decision boundary between viable and non-viable embryos based on those mechanical parameters, and performed feature selection to determine that three parameters (again *k*_1_, *η*_1_ and *k*_0_) provided optimal predictive value (Supplementary Fig. 2C). Receiver operating characteristic (ROC) and precision-recall (PR) curves are shown in Supplementary Fig. 2A,B. When using our classifier to predict human embryo blastocyst formation based on their mechanical properties, the area under the ROC curve is 0.87 and the area under the PR curve is 0.86, which corresponds to >90% precision, 95% specificity and 75% sensitivity.

Once we determined that our mechanical parameters could reliably predict viability at the zygote stage, we compared their performance to other parameters that are commonly used to predict embryo viability at an early stage. For all 89 human zygotes, we extracted cell cycle parameters by measuring time intervals between the key cleavage stages (Supplementary Fig. 3A,B). We observed that time (in hours) (i) of duration of first cytokinesis (*c*_1_), (ii) between the first and second mitosis (*c*_2_) and (iii) between second and third mitoses (*c*_3_) were (*c*_1_=0.20, *c*_2_=11.32, *c*_3_=2.91) for embryos that developed to the blastocyst stage (31 out of 89), which overlaps with previous reports^27^. Embryos that arrested had a widely varying set of parameters. When these three parameters were used to predict blastocyst formation, the sensitivity and specificity were 85% and 88%, respectively, with AUC_ROC_ of 0.95 and AUC_PR_ of 0.91. Although mechanical parameters alone have slightly lower predictive power compared with cell cycle parameters at the four-cell stage, they currently represent the most effective method of predicting viability before the embryo reaches three to four cells. In particular, at the zygote stage, only mechanical parameters can provide information about embryo viability, and an additional 48 h in culture is required for time-lapse parameters to surpass their predictive power (Supplementary Fig. 3D). Mechanical parameters can also improve on the predictive value of cell cycle parameters alone (Supplementary Fig. 2A,B); the optimal combination of mechanical and cell cycle parameters (*k*_1_, *η*_1_, *c*_1_ and *c*_2_) can achieve a sensitivity and specificity of 90 and 91%, with AUC_ROC_ of 0.97 and AUC_PR_ of 0.94 (Supplementary Fig. 2D, four-cell stage).

### Zygote mechanical parameters also predict live birth in mouse

We investigated whether embryo mechanical properties were predictive of development to term *in vivo* in addition to blastocyst formation *in vitro* using mouse as a model. A classifier was first made for predicting mouse embryo viability as it was made for the human embryos. Briefly, we measured the mechanical properties of a total of 282 mouse zygotes at the 2PN stage and then cultured them *in vitro* to define good or poor developmental potential based on blastocyst development. Only embryos that were fertilized with two polar bodies were selected for the measurement. A scatter plot of the three most important parameters is shown in Supplementary Fig. 4. Embryos for which we measured mechanical properties did not have significantly different blastocyst formation rates (67%) than control embryos (70%) (Supplementary Fig. 5), suggesting that the measurement does not lower mouse embryo viability (*P*=0.62, two-proportion z-test). As with the human embryos, mouse embryos that reached the blastocyst stage occupied one region of the scatter plot around (*k*_1_=0.17 N m^−1^, *η*_1_=1.3 N s m^−1^ and *k*_0_=0.06 N m^−1^), while non-viable embryos were scattered away from the ‘average' viable embryo. We trained an SVM classifier on the mouse data and found that mechanical parameters could predict blastocyst formation by the zygote stage with AUC_ROC_ of 0.85 and AUC_PR_ of 0.92, which corresponds to a sensitivity of 76% and a specificity of 79%.

After the classifier was validated, the mechanical parameters of 204 mouse zygotes were measured and grouped according to their mechanical properties. A total of 55 viable and 55 non-viable embryos were transferred to recipient female mice within 2 h after the measurement and analysis, so that each mouse received 12–15 embryos, which were either all viable or all non-viable. The results of the experiment are shown broken down by mouse and experiment (four experiments total) in Supplementary Table 1 and as a whole in Fig. 2g. Embryos classified as viable based on mechanics were significantly (*P*<10^−6^, *χ*^2^-test) more likely to result in a live birth (74%) compared with embryos classified as non-viable based on mechanics (24%), and they were also significantly (*P*=0.01, *χ*^2^-test) more likely to result in a live birth than control embryos selected by morphology (49%). This indicates that mechanical parameters are a true measure of ultimate survival potential in mouse, and our measurement does not affect embryo development. Although the causes of embryonic arrest may differ between mice and humans, and thus results in a mouse model may not always translate to humans, we find these results encouraging. Future studies will reveal whether mechanical properties of human embryos can also predict clinical pregnancy and live birth.

### Human zygote gene expression correlates with viability

**Figure 3:**
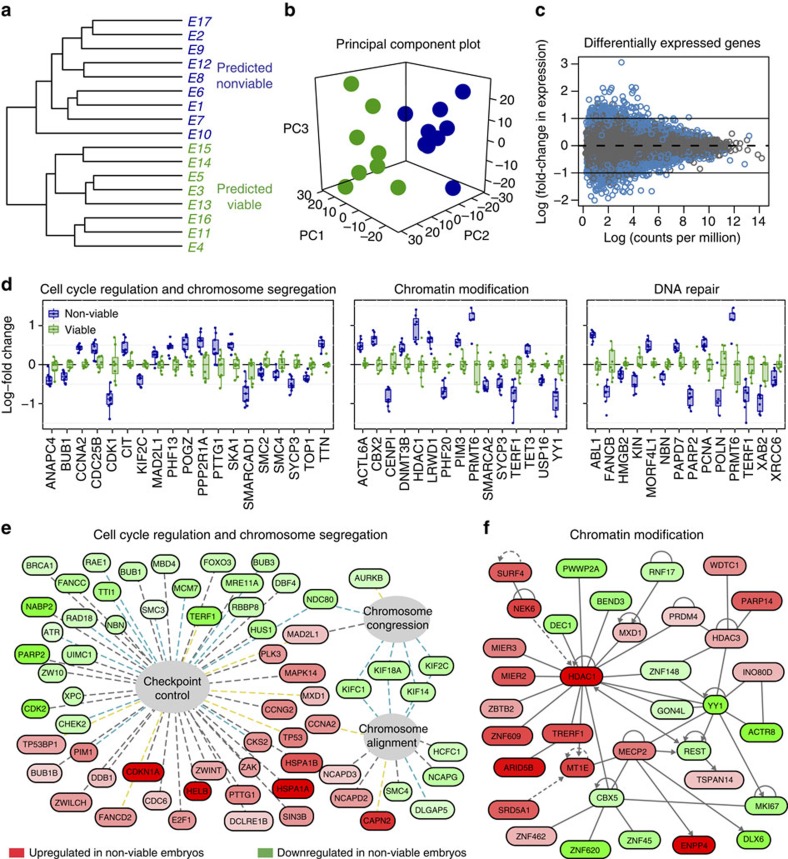
Results of RNA-seq on eight predicted viable and nine predicted non-viable human zygotes (predicted based on mechanics). (**a**) Hierarchical clustering of gene expression values show that zygotes cluster by viability. *N*=8 viable and *n*=9 non-viable zygotes were used over a total of two replicates. (**b**) 3D principal component plot also shows that zygotes cluster by viability. (**c**) Using the edgeR package in R, we found 2,522 genes with statistically significant (*q*-value<0.01) differences in expression between viable and non-viable embryos (blue circles), most of which had a log-fold change of at least 0.5. (**d**) We found differences in many gene categories important in oocyte maturation and fertilization. Box plots containing the median and IQR of expression of particularly interesting genes are shown here. Whiskers extend up to 1.5 times the IQR from the box edges as in Fig. 2d. (**e**) IPA predicted a significant decrease in cell cycle checkpoint control and chromosome segregation in non-viable embryos (*P*<0.01). (**f**) A network of genes predicted to interact with each other (*P*<0.01), which are involved in chromatin modification, a process important for the oocyte-to-embryo transition.

After validating the predictive power of our mechanical parameters, we asked whether developmental potential is reflected at the level of transcription, and, if so, what patterns of gene expression are characteristic of a viable or non-viable zygote. We measured the mechanical parameters of 22 previously frozen human zygotes at the 2PN stage, which were classified as viable or non-viable based on their mechanical parameters. Only embryos with another ‘sibling' embryo were chosen for sequencing to account for interpatient variability in post processing. Single-cell RNA-sequencing (RNA-seq) on the 17 remaining single embryos generated 441-Gb data with 31 million paired-end reads per embryo (on average) with read length of 100 bp. We detected expression of 12,342 genes, of which 1,879 (15%) were differentially expressed (DE) with a false discovery rate (or *q*-value) below 1%. All ‘significant' differences in gene expression have *q*-value<0.01 as output by the edgeR^28^ package in R. Figure 3a shows hierarchical clustering of gene expression data after removal of batch effects. As expected, embryos cluster by predicted viability. Figure 3b shows a plot of the top three principal components, capturing 90% of the variation in the data set. Again, viable and non-viable embryos appear well separated, with the first principal component capturing the majority of the variation. The dendrogram and principal component plot were both made based on all genes with expression values greater than 0.5 counts per million. Figure 3c shows a scatter plot of the log-fold change in expression between viable and non-viable embryos plotted against the log of the expression counts per million (c.p.m.) for each gene.

We further examined the RNA-seq data to determine which biological processes (BPs) may have been affected by the DE genes, and to investigate why non-viable embryos were destined to arrest. We used the Database for Annotation, Visualization, and Integrated Discovery (DAVID) tool to perform gene ontology clustering on our list of DE genes and determine whether any molecular functions or BPs were over-represented. Table 1 shows a list of the 19 functional annotation clusters with at least one component in each cluster containing an adjusted *P* value (*q*-value) below 0.05, along with its enrichment score. We then used Ingenuity Pathway Analysis (IPA) to examine the predicted biological effects of the gene expression differences seen between viable and non-viable embryos, and to identify networks of co-regulated genes.

Clusters 4, 10 and 17 in Table 1 refer to processes involved in nuclear maturation. Figure 3d contains box plots of selected genes from these clusters that are DE between viable and non-viable embryos. In particular, we found that non-viable embryos have reduced expression of genes including a cyclin-dependent kinase (*CDK1*), which is essential for mitotic cell cycle regulation in mammals^29^, germinal vesicle breakdown and meiosis resumption^30^, and was expressed twofold lower in non-viable embryos. Concurrently, we found a 1.3-fold difference in the expression of *CDC25B*, which is indispensable for *CDK1* activation. We also found changes in expression of many cyclins and other cyclin-dependent kinases, including *CDK2, CDK4* and *CCNA2*, which are all important in regulating cell cycle and oocyte maturation. Our study also revealed differences in expression of 73 genes involved in chromosome segregation and ploidy status regulation. We found differences in expression of *BUB1, BUB1B, BUB3, MAD2L1*, securin (*PTTG1* and *PTTG2*), members of the anaphase-promoting complex (*ANAPC1, ANAPC4* and *ANAPC11*), structural maintenance of chromosome proteins *(SMC2, SMC3* and *SMC4*) and DNA topoisomerases *(TOP1*, *TOP2A* and *TOP2B)*. A meiotic marker gene *SYCP3* was also shown to be DE in non-viable embryos.

We then used IPA to predict the effect of our patterns of differential expression on cell cycle progression and aneuploidy prevention. The results of this analysis (Fig. 3e) indicate that non-viable embryos have a reduced ability to carry out cell cycle checkpoint control, chromosome alignment and chromosome congression (blue lines indicate that expression of that gene agrees with the predicted effect and yellow lines indicate that it disagrees).

Clusters 1, 7, 12 and 18 in Table 1 are related to epigenetic modification of chromosomes and regulation of gene transcription. We found significant differences in expression of 161 genes involved in chromatin remodelling, some of which are plotted in Fig. 3d. Specifically, we found differences in expression of *DNMT3B*, *HDAC1, TET3* and *YY1*. Loss of any of these genes results in embryonic lethality or a higher rate of developmental failure due to aberrant epigenetic modifications^31^,^32^,^33^,^34^. Figure 3f shows an interaction network (*P*<10^−32^) calculated with IPA that includes genes involved in chromatin modification, coloured by the log-fold change between viable and non-viable embryos. IPA analysis also predicted that chromatin organization and modification activity was reduced in non-viable embryos based on the log-fold change in gene expression we found between viable and non-viable embryos (Supplementary Fig. 6).

Cluster 2 in Table 1 refers to the embryo's ability to repair its DNA, implying that DNA damage may be another possible mechanism of embryonic arrest. We found differences in expression of many genes involved in DNA repair and telomere maintenance, including *BRCA1, TERF1, ERCC1, XRCC6, XAB2, RPA1* and *MRE11A*. IPA analysis on our data also found that DNA repair was reduced in our non-viable embryos (Supplementary Fig. 7), and that non-viable embryos are more likely to have poor blastocyst morphology (Supplementary Fig. 8).

Clusters 3, 8, 11, 14, 16 and 19 in Table 1 refer to protein synthesis, modification, localization and degradation, all of which are vital to proper cytoplasmic maturation in oocytes and proper development after fertilization. Of these, several (*ATG5, CUL3, USP11, USP2* and *UBE2G2)* are known to play vital roles in protein ubiquitination and autophagy, and loss of function is association with pre-implantation arrest and embryonic lethality^35^,^36^,^37^. These results reinforce that idea that timely degradation of maternal transcripts is vital for ensuring that embryos can develop successfully after EGA.

### Non-viable oocytes may undergo suboptimal fertilization

**Figure 4:**
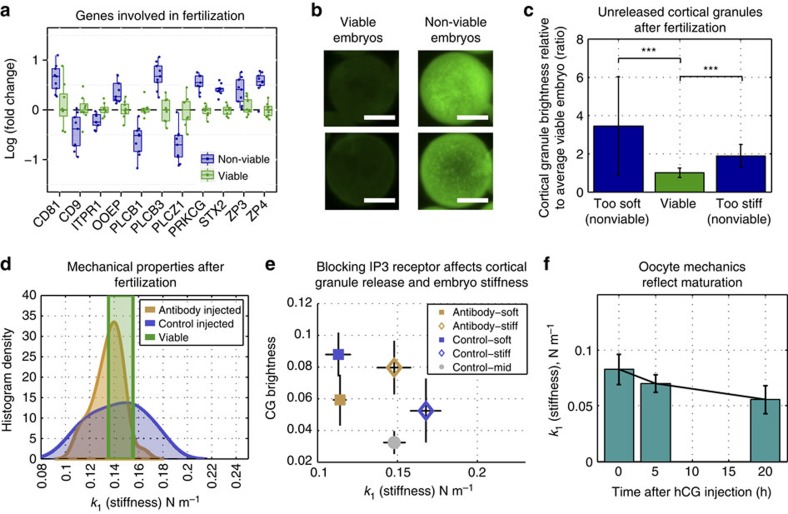
Cortical granule staining reveals differences between fertilization between predicted viable and non-viable embryos based on mechanics. (**a**) List of some of the genes important for fertilization identified as differentially expressed in RNA-seq results. (**b**) Representative images of mouse embryos stained for cortical granules. (**c**) Non-viable embryos have higher signal from cortical granules, regardless of whether they are too soft (*n*=24, *P*<10e−6, Wilcoxon rank sum test) or too stiff (*n*=13, *P*<10e−5, Wilcoxon rank sum test) compared with viable embryos (*n*=30). ***P<0.001. (**d**) Blocking of IP_3_ receptor in oocytes (*n*=32) before fertilization resulted in slightly softer mechanics after fertilization compared with control embryos (*n*=19), and absence of very stiff embryos. (**e**) Stiffest quartile of antibody-injected embryos showed lowered stiffness after IVF compared with stiffest quartile of control-injected embryos (*P*=0.02, Wilcoxon rank sum test), and increased brightness (arbitrary units) from unreleased cortical granules compared with control embryos with similar mechanical properties (*P*<0.01, Wilcoxon rank sum test). (**f**) Oocytes become less stiff (*P*<10e−7, Wilcoxon rank sum test) over the course of maturation from the GV (*n*=35) to MI (*n*=49) to MII (*n*=65) stages. Error bars represent s.d.. Scale bar, 25 μm.

Our human RNA-seq data indicated that there may be differential expression of many genes important for fertilization between viable and non-viable embryos (Fig. 4a). Of these, *CD9, ZP3* and *ZP4* are found on the egg's plasma membrane and in its zona pellucida (ZP), and could inhibit sperm–egg binding if expressed incorrectly. Interestingly, we found reduced levels of *PLCZ1*, an mRNA that codes for a sperm protein, in the non-viable zygotes. We also found reduced expression in non-viable zygotes of the IP_3_ receptor *ITPR1*, which is important for initiating the Ca^2+^ oscillations that lead to cortical granule release and zona-hardening.

To test whether the extent of cortical granule exocytosis accounts for differences in embryo mechanical properties, we classified mouse zygotes as viable or non-viable based on their mechanical properties after IVF, and then imaged the cortical granules remaining within the cytoplasm using confocal microscopy. All of the viable zygotes (*n*=30) had low levels of signal, while most of the non-viable zygotes (*n*=37) had very bright signal (Fig. 4b). Using a Wilcoxon rank sum test, we found that the median value of this intensity profile was much higher in non-viable embryos, regardless of whether they had lower (*n*=24, *P*<10^−6^) or higher stiffness (*n*=13, *P*<10^−5^) than viable embryos (*n*=30; Fig. 4c). These results suggest that cortical granule exocytosis can influence embryo stiffness but there are likely other factors that also explain the link between embryo mechanics and viability.

We next microinjected oocytes (*n*=32) with an antibody to the IP_3_ receptor (*ITPR1*) to block cortical granule release and observed changes in embryo stiffness after fertilization. We also performed sham injections on 19 control embryos. Microinjection did not inhibit sperm penetration, as two pronuclei could be observed in 37% of the injected and 40% of noninjected oocytes after insemination. However, introduction of the antibody lowered embryo stiffness from 0.143±0.022 N m^−1^ for control embryos to 0.135±0.012 N m^−1^ (*P*=0.12, Wilcoxon rank sum test; Fig. 4d). Although injection of the antibody did not induce a statistically significant change in the mean stiffness (two-sided *t*-test), there was a statistically significant change in the distribution of stiffness values (*P*=0.03, two-sample Kolmogorov–Smirnov test). The stiffest quartile of antibody-injected embryos was significantly softer (*P*=0.03, Wilcoxon rank sum test) and had significantly higher signal from cortical granules (*P*=0.03, Wilcoxon rank sum test) compared with the stiffest quartile of control-injected embryos, indicating less cortical granule release (Fig. 4e). They also exhibited significantly less cortical granule release than would be expected for a group of embryos with similar mechanical parameters (*P*=0.008, Wilcoxon rank sum test; Fig. 4e). The softest quartile of antibody-injected embryos appeared unaffected, as they did not exhibit significantly different stiffness (*P*=0.73, Wilcoxon rank sum test) and cortical granule release (*P*=0.06, Wilcoxon rank sum test) from the softest quartile of control embryos (Fig. 4e).

We also found that oocyte stiffness falls significantly over the course of maturation (*P*<10^−5^ from GV to MI and *P*<10^−9^ from MI to MII, Wilcoxon rank sum test). As oocytes mature, they become softer; average oocyte stiffness is 0.083±0.013 N m^−1^ at the GV stage (n=35), 0.070±0.008 N m^−1^ at the MI stage (*n*=49) and 0.056±0.013 N m^−1^ at the MII stage (*n*=65; Fig. 4f). Approximately 5–6 h elapsed between collection of GV and MI oocytes, and 12–15 h elapsed between collection of MI and MII oocytes, indicating that oocytes undergo a rapid softening after germinal vesicle breakdown, which slows down between the MI and MII stages. Our results imply that non-viable embryos exhibit a diverse array of mechanical phenotypes that may reflect a reduced capacity to complete embryogenesis. These differences in mechanics may be caused by abnormalities in oocyte-softening and maturation before fertilization, or in the ability to undergo cortical granule exocytosis at the time of fertilization.

## Discussion

**Figure 5:**
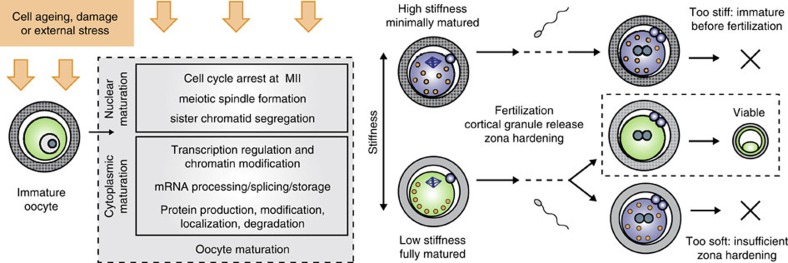
Model for how embryo fate is determined and why it is detectable mechanically. Some oocytes fail to achieve optimal maturation because of external stress during maturation, or inherent poor quality. At the time of fertilization, these oocytes (blue cytoplasm) may be overly stiff and still immature, or they could fail to release cortical granules properly and be overly soft after fertilization. Optimally matured oocytes (green cytoplasm) will release cortical granules into the perivitelline space, undergo the appropriate changes in mechanical properties and go on to develop successfully.

For the first time, we have demonstrated a technique to accurately assess human embryo viability at the 2PN stage, while the zygotic genome is transcriptionally inactive and development is still controlled by maternal mRNAs and proteins. The ability to predict embryo viability at such an early stage strongly suggests that much of human embryo developmental potential is determined before fertilization by the contents of the oocyte. Although DNA fragmentation or damage in the sperm can lower a healthy oocyte's developmental potential, it is the oocyte that contributes the vast majority of the cytoplasmic contents, which are also vital for successful development. A proposed model of embryo viability determination is shown in Fig. 5.

Many previous studies have focused on oocyte and embryo-quality assessment, as well as understanding how embryo fate is determined. In particular, it has been suggested that the first two embryonic cell divisions are primarily controlled by maternal genes^38^ and that, in the mouse, the first division in embryos affects subsequent development^39^. Knockout experiments in mouse oocytes such as *stella* and *Hsf1* have allowed identification of oocyte genes, playing an important role in embryo development^40^,^41^. Most recently, RNA-seq on single human embryos revealed stage-specific transcripts and provided a landscape of candidates that are important in mammalian pre-implantation development^27^,^42^,^43^; however, very little zygote data have been available. Our data provide a global view of differences in the transcriptomes of zygotes of high and low developmental potential.

We found that non-viable embryos exhibited significantly different expression of genes controlling cell cycle progression, including nearly all of the cyclin-dependent kinases and many of the cyclins. These differences imply that non-viable embryos may have trouble resuming their cell cycles following fertilization and may help to explain why the timing of the first cell division can predict blastocyst formation^27^,^44^. We also found that non-viable embryos misexpress genes important in sister chromatid cohesion, alignment or segregation, or in the spindle assembly checkpoint, which may help explain the high levels of aneuploidy seen in arrested human embryos^45^. Our data also indicate that, much like aged oocytes, non-viable zygotes have significant deficiencies in DNA repair and telomere maintenance. Interestingly, even though we removed interpatient variation in our data, we still found patterns of gene expression in our non-viable zygotes that were similar to those of aged oocytes^46^,^47^. Our results suggest that there is intrapatient heterogeneity in viability, where non-viable zygotes may have ‘aged' faster than viable zygotes.

Our results also suggest that non-viable zygotes also have deficiencies in cytoplasmic maturation, which may impair their ability to switch from maternal to zygotic control and develop successfully after fertilization. While nuclear maturation is defined by cell cycle progression and can be easily evaluated on a simple observation, the factors that define successful cytoplasmic maturation are still poorly characterized because of the lack of an early predictor of viability. Reverse transcriptase–PCR-based and microarray-based analyses on maternal transcripts have identified a number of genes that are expressed in oocytes and are important for oocyte development and maturation^48^,^49^. This study helps to better define cytoplasmic maturation at the transcriptome level, and represents the first insight into how maternally inherited gene expression patterns correlate with developmental potential in zygotes originating from MII oocytes.

Our RNA-seq data also revealed that non-viable embryos misexpress several genes involved in fertilization, which could impair cortical granule release and zona-hardening, thus affecting the resulting embryo's mechanical phenotype. In particular, non-viable embryos show reduced expression of *ITPR1*, a receptor that is involved in the signalling cascade initiated by fertilization, and that shows an increased expression during oocyte maturation. Insufficient levels of *ITPR1* expression may affect oocyte activation, cortical granule release and, therefore, embryo mechanical properties. Our data suggest that there may be at least two mechanisms for non-viable embryos to develop different mechanical properties from viable embryos. The first may be a failure to release cortical granules into the perivitelline space and undergo sufficient zona-hardening, possibly caused by misexpression of genes upstream in the fertilization-signalling pathway as seen in the RNA-seq results. The second may be a failure in achieving appropriate maturation before fertilization, causing an overly stiff oocyte to become an overly stiff embryo. These results suggest that embryo mechanical properties could provide us with insight into the quality of oocyte maturation and the resulting embryo's developmental competence. Because mouse and human embryos exhibit differences in cortical granule synthesis and distribution^50^, follow-up studies should be performed in human oocytes to understand the link between oocyte maturation, cortical granule release and embryo mechanical properties. In the IVF clinic today, oocyte maturation is assessed simply by looking for the lack of a germinal vesicle and the presence of a polar body. Because we found that oocyte mechanical properties change over the course of maturation, further work in this area could yield a minimally invasive measure of oocyte maturation with finer resolution than is possible through a morphological assessment alone.

The micropipette aspiration approach we used in this study was simple and effective in measuring four mechanical parameters, of which three were predictive of embryo viability. One limitation of this approach is its reliance on a bulk model of the embryo as a whole, which lacks an exact correspondence between model parameters and embryo components. If finer spatial resolution in measuring mechanical properties is desired, an alternate approach should be used or more outputs should be measured in addition to the cell aspiration depth into the micropipette. Another potential limitation of our approach is the potentially large (∼30%) deformation applied to the embryo. Although larger deformations are routinely applied during ICSI, studies have shown that applying mechanical stress to cells can alter levels of gene expression^51^,^52^, induce actin polymerization^53^ and even affect viability^54^. The mouse embryos we measured were only deformed for a few seconds, did not exhibit lower blastocyst formation rates and seemed to recover to their original shape within several minutes; however, the high amount of deformation could still have unknown effects on gene expression and future development.

The modified Zener model used in this paper represents a bulk measurement of the mechanical properties of the embryo as a whole. Although the 2PN embryo is a mechanically heterogeneous structure, we can estimate its Young's modulus to be ∼0.5 kPa for mouse embryos using equation (5) from a review on micropipette aspiration^25^. This value is similar to that of cells that lack a ZP but exhibit solid-like behaviour such as endothelial cells and chondrocytes^24^,^55^. Some recent studies have focused on measuring the Young's modulus of 2PN embryo ZP and obtained values around 20–40 kPa (refs 18, 22, 56, 57) in mouse and 84 kPa (ref. 58) in cow. Taken together, these results suggest that the embryo is a mechanically heterogeneous structure consisting of a relatively soft cell surrounded by a stiff ZP. Future studies could investigate the relative contributions of cell and ZP mechanics to bulk embryo mechanics and developmental potential.

Although all of the mouse embryos used in this study were fresh, the human embryos were previously subjected to cryopreservation, which has been shown to affect zona stiffness^59^ and cortical granule exocytosis^60^. In our own data, we found that cryopreservation does cause an immediate increase in embryo stiffness, but this effect disappears by 3 h post thaw (Supplementary Fig. 9). Therefore, to eliminate a potential confounding effect due to cryopreservation, we allowed all human zygotes to recover in the incubator for 3–4 h after thawing. It has also been suggested that ICSI and *in vitro* culture conditions can affect cortical granule exocytosis and embryo mechanical properties^61^,^62^. In this study we did not have access to information on the fertilization method (ICSI/IVF), or culture conditions before freezing, which may have lowered the predictive power of our human zygote viability classifier.

One of the advantages of our approach to predict embryo viability is the applicability of our model to both mouse and human embryos, despite their numerous differences. In addition to lacking the same propensity for chromosome abnormalities as human embryos, mouse embryos are different mechanically as well; they have a much thinner ZP for their size, and they have a high oolemma elasticity that makes ICSI challenging^63^. In our data, we found that mouse embryos have much lower stiffness and higher viscosity than human embryos, which may help explain why ICSI is so difficult in the mouse.

Some limitations of using SVM to construct a classifier are its sensitivity to the box constraint *c* and the radial basis function (RBF) parameter *σ*, and its need for large amounts of training data for high-dimensional problems. Choosing the wrong values for *c* and σ could lead to underfitting the data and obtaining poor classification accuracy, or overfitting the data and being overly sensitive to outliers. To avoid these scenarios, we employed an optimization algorithm using 10-fold cross-validation to choose parameter values that maximized classification accuracy and avoided overfitting. In this study high dimensionality was not a problem; however, if our model were expanded to include more parameters, many more embryos would have to be measured to effectively train a classifier. After proper parameter tuning and training, our classifier performed quite well; although the distributions of viable versus non-viable embryos differ between mouse and human data (Fig. 2e and Supplementary Fig. 4), we found our classifier to be highly accurate and adaptable to both data sets because of the use of the RBF kernel. The flexibility of our machine-learning approach indicates that our classifier could be applied across multiple different species, or could be further augmented with mechanical data from oocytes or fresh human embryos.

The ability to accurately predict an embryo's viability directly after fertilization also has immediate applications in the IVF clinic. Traditionally, embryo selection has consisted of a morphological assessment, which is highly subjective. The development of non-invasive time-lapse imaging and blastocyst culture techniques has helped to improve embryo selection and reduce the average number of embryos transferred. However, although cell cycle parameters are slightly more effective at predicting blastocyst formation compared with mechanical parameters, extended time in culture is stressful to embryos and can negatively have an impact on epigenetic reprogramming^64^,^65^. The mechanical parameters we have developed promise to further improve the clinical practice of IVF because they can be measured in a fully automated and minimally invasive manner, as soon as fertilization is complete. In the short term, they could be used to add predictive value to current embryo selection techniques such as cell cycle parameters and allow clinicians to more confidently transfer a single embryo. However, after further validation, we expect that they could enable the transfer of embryos directly after fertilization, allowing embryos to begin development in their natural environment. Because mechanical parameters can be measured at any stage of development, our work also opens the door to better assessments of oocyte quality and maturation, which could improve oocyte culture, handling and fertilization rates in the clinic.

## Methods

### Human sample source

A total of 133 2PN-stage human embryos used in the current study were obtained from the Stanford University RENEW Biobank, with written informed consent that they were donated for non-stem research. All embryos were frozen at the zygotic or 2PN stage, and it is very likely that these embryos represent the typical IVF population, since they were not selected before cryopreservation. De-identification was conducted in compliance with the protocol ‘The RENEW Biobank', which was approved by the Stanford University Institutional Review Board. No protected health information was associated with the embryos. Of the 133 embryos thawed, 91 appeared alive after thawing, and the rest were excluded because they were visibly not viable (before any micropipette aspiration). Two more embryos were excluded from our data because they were structurally damaged during micropipette aspiration and because of ZP rupture, leaving us with 89 total human embryos in our data set. These 89 human embryos were thawed and measured in smaller groups over the course of six separate experiments.

### Human embryo culture

Human embryos were thawed using a Quinn's Advantage Thaw Kit (CooperSurgical) as described before^26^,^27^. In brief, cryovials or straws were removed from the liquid nitrogen tank and exposed to air before being placed in a 37°C water bath. Once thawed, embryos were incubated in 0.5 and 0.2 M sucrose thawing medium at 37 °C for 10 min each. The embryos were then washed in thaw diluent solution at 37 °C and cultured for 3–4 h in Quinn's advantage cleavage medium (CooperSurgical) supplemented with 10% serum protein substitute (CooperSurgical). In some experiments, embryos were monitored for their development using the Eeva System (Auxogyn).

### Mouse embryo and oocyte collection and IVF

Hybrid strain (C57B6xDBA2) F1 mice (4–6-week-old females) were purchased from Jackson Laboratory and maintained in the animal facility in the Lorry Lokey Stem Cell Research Building. All experiments were performed under Institutional Animal Care and Use Committee protocol #16146, which was approved by the Administrative Panel on Laboratory Animal Care at Stanford University. Briefly, female mice were superovulated by intraperitoneal injection of 10IU pregnant mare serum gonadotrophin (PMSG, Sigma) 10IU human chorionic gonadotrophin (hCG, Sigma). After mating one female with an 8–10-week-old male of the same strain, cumulus–oocyte complexes were collected from oviducts into M2 media (Millipore) and cumulus cells were removed in Hyaluronidase (Sigma) and washed in M2 media. After mechanical measurements, the embryos were cultured in Quinn's advantage cleavage medium (CooperSurgical) with 10% serum protein substitute (CooperSurgical). To measure the effect of oocyte maturation on mechanics, oocytes were collected at various stages of maturation. GV oocytes were collected 48 h after injection of PMSG. Approximately 48 h after PMSG injection, hCG was injected. MI oocytes were collected 5–6 h after hCG injection, and MII oocytes were collected 18–20 h after hCG injection.

Embryos were excluded from our data if they showed very poor morphology, if they came from mice that produced fewer than 10 embryos or if they were in a group where fewer than 40% of embryos showed pronucleus formation after IVF. If the mice produced too few embryos or fertilization rates were too low, we deemed that some parts of the experimental protocol (hormone injection, media, and so on) must have negatively affected the embryos and thus they might not be representative of normal mouse embryos. To sort embryos into control and measurement groups, they were randomly separated while being viewed under low enough magnification that morphology was difficult to distinguish. Only a few embryos were kept as negative controls in each an experiment, summing to a total of 35 over all experiments (Supplementary Fig. 5). Sample sizes for animal experiments were determined in part by the amount of data needed to reach statistical significance, and in part by sample sizes seen in publications with similar research. Mechanical and blastocyst formation data for the 282 mouse embryos we measured (Supplementary Fig. 4 and Supplementary Fig. 5) were collected over the course of 15 separate experiments.

### Microinjection of mouse oocytes and IVF

A CBA mouse strain from Jackson Laboratory was used in this experiment. After induced superovulation of 4–6-week-old female mice, metaphase-II-arrested oocytes were collected and cumulus cells removed. Oocytes were then microinjected with ∼10 pl of monoclonal antibody 18A10 (abCam) at a concentration of 1 mg ml^−1^. Some oocytes were kept as negative controls and underwent either sham injection with water or no injection at all. The oocytes were then cultured for an hour before conventional IVF. Sperms were collected from 8–10-week-old CBA males (Jackson Laboratory) and allowed to capacitate in HTF media (CooperSurgical) for 45 min before insemination. An appropriate amount of the sperm suspension was added to the media drop where oocytes were cultured. No samples were excluded from this experiment, and oocytes were sorted into control versus measured groups without regard for morphology and in a random order.

### Measurement of embryo mechanical properties

An automated micropipette aspiration system was used to measure the embryos, and a custom-built light microscope was used to record video of the aspiration. The light microscope was built with parts from Thorlabs, and it uses a × 20, 0.4 numerical aperture Olympus objective and white light-emitting diode illumination. To conduct a measurement, each embryo is placed in its own drop of media and the micropipette is lowered into the drop. The embryo is manipulated with the pipette so that the polar body is facing away from the pipette opening. Failing to control for the measurement position could confound our results, since there is a space between the oolemma and ZP close to the polar body, while this perivitelline space is much smaller around the rest of the embryo. Studies have also shown that oocyte cortical tension is polarized with respect to the meiotic spindle^66^; therefore, we took care to measure the end of the embryo opposite the polar body, which is usually close to the spindle.

Micropipettes for measuring mouse embryos had 40-μm inner diameter (Origio MBB-FP-L-15); those for human embryos had 70-μ inner diameter and were custom-made by Origio. A holding pressure of −0.03 p.s.i. is applied to hold the embryo and seal the pipette opening. Then, a step pressure of −0.345 p.s.i. is applied to the embryo. The desired pressure is applied using a closed-loop PID control system. A linear actuator (Firgelli L12) moves the stopper of a 1-ml syringe, and a sensor (Honeywell SSCSNBN010NDAA5) measures the pressure. These are connected to a computer via an Arduino Uno microcontroller that can relay sensor readings and give commands to the actuator in real time. A video of the aspiration is recorded at 75 f.p.s. using a CMOS camera (Thorlabs DCC1545M). All hardware are controlled using the custom-made Labview software (National Instruments). An automated custom-made Matlab programme (Mathworks) was used to extract the aspiration depth of embryo and fit it to a mechanical model. Canny edge detection and thresholding were used to identify pipette corner and opening. Cross-correlation-based template matching was used to automatically track embryo edge over time to remove bias from manual measurements. During the measurement of embryo mechanical parameters, the investigator was blinded to whether they survived to the blastocyst stage or not, or to which group they belonged (in the case of the microinjection experiments).

### Mechanical model of one-cell embryos

A modified linear elastic solid model (modified Zener model) was used to represent the embryo as it was aspirated into the micropipette. Assuming a step input for the force applied to the embryo, the equation for aspiration depth over time is:


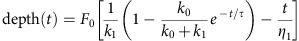


Where


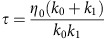


The modified Zener model was chosen over other commonly used viscoelastic models such as the Maxwell, Kelvin-Voigt and Zener (Standard Linear Solid) based on visual inspection of the response dynamics. We observed in our experiments that an embryo's response to a step pressure input was an instant elongation followed by an exponential decay component (inverted so the deformation increased over time), finally followed by deformation at a constant rate as shown in Fig. 2c.

A Maxwell body consists of a spring and dashpot in series; therefore, its response to a step pressure is an instant elongation followed by a steady linear deformation. Because our data displayed a clear exponential component, this model was deemed insufficient to describe our system. A Kelvin-Voigt body consists of a spring and dashpot in parallel; therefore, its response to a step pressure is a decaying exponential where the aspiration depth settles on a final maximum depth. This model fails to capture the instant elongation experienced by the embryo as well as the linear rate of deformation after the exponential component settles. The Zener model adds an extra spring in parallel to the Maxwell body and its response to pressure is similar to that of a Kelvin-Voigt body, but with an instant elongation when the pressure is first applied. Still, this model required the addition of an extra dashpot in series with the other three components to accurately capture the linear deformation experienced by the embryo after the exponential component settled.

A comparison of the fitting error for five different models is shown in Supplementary Fig. 10. Our model (which has 4 parameters) appears appropriate because its error is nearly as low as the error of the five-parameter model, with or without the unnecessary extra parameter. We therefore believe our model accurately describes the data without overfitting.

### Assessment of embryo viability and cell cycle parameters

Blastocyst formation was used as a proxy for viability when constructing a classifier to distinguish between viable and non-viable embryos based on mechanics. This classifier was validated in mouse as shown by the live birth experiments in Fig. 2. After embryo mechanical parameters were measured on day 1, embryos were cultured for 5–6 days inside the Auxogyn Eeva system, with frames captured every 5 min. Cell cycle parameters^27^ were extracted from the resulting videos, and embryos forming blastocysts by day 5 (mouse) or day 6 (human) were categorized as viable.

### Embryo viability prediction

Blastocyst survival was used as ground truth information for training a classifier to predict embryo viability based on day 1 mechanical measurements. A binary SVM classifier with a RBF kernel was trained on embryo mechanical parameters. Optimal values for the box constraint (*c*) and RBF sigma (*σ*) were chosen using 10-fold cross-validation. Once the optimal SVM parameters were determined, 100 Monte Carlo simulations of 10-fold cross-validation were performed to calculate the average area under the ROC curve and PR curve.

Because a total of four mechanical parameters were measured for each embryo, forward feature selection was conducted to determine the optimal number of parameters to include in our viability classifier. The results of this process are shown in Supplementary Fig. 2C, demonstrating that there is a large improvement when using two parameters instead of one, and a slight improvement when adding a third parameter. Using all four parameters reduces the effectiveness of our classifier because the fourth parameter is not useful in separating embryos by viability but adds an extra dimension to the data.

To conduct forward feature selection, first each parameter was used on its own to separate embryos and an optimal classifier was found. As described above, 10-fold cross-validation was conducted to estimate the area under the ROC curve for that parameter. Once the parameter with the highest predictive value was chosen, a new classifier was trained and optimized using each of the remaining parameters. The parameter that caused the highest improvement in predictive value was added to the classifier, and the process was repeated until there were no parameters remaining.

For the experiments on cortical granule release, figures were shown using only the parameter most predictive of viability (stiffness, *k*_1_) for simplicity. The previously trained three-parameter classifier was used to sort embryos by viability, but we determined the figures to be clearest when using stiffness only.

### Mouse live birth experiments

CD1 female mice were purchased from Jackson Laboratory and mated with vasectomized males to serve as pseudopregnant recipients. Mice were kept at the same condition as those used for embryo collection. The mechanical parameters of embryos collected from (C57B6xDBA2) F1 mice were measured and the embryos were sorted into viable or non-viable groups based on these parameters. In each experiment, an equal number of one-cell stage mouse embryos (range from 10 to 15) were transferred into the oviduct of a recently plugged embryo recipients. This experiment was repeated four times (Supplementary Table 1) with 10–15 embryos transferred to each mouse in each experiment. The technician transferring the embryos was blinded to which embryos were in which group. The same number of embryos at the same developmental stage were randomly chosen and transferred to pseudopregnant mice as a control. The pups were expected on day 20 following surgery. A *χ*^2^-test was performed to test whether the difference in number of pups born in each group (viable, non-viable or control) was statistically significant.

### RNA-seq sample preparation

Two separate experiments were conducted with a total of 32 2PN human embryos to obtain samples for RNA-seq. The embryos were allowed to recover for 3–4 h in culturing media after thawing and the mechanical parameters were measured. Each embryo was classified as viable or non-viable based on its mechanical parameters. cDNA from each embryo was synthesized from total RNA using the SMARTer Ultra Low RNA kit (Clontech) according to the provider's protocol. After Covaris shearing of full-length cDNA, final libraries were generated using the NEBNext DNA Sample Prep Master Mix Set (New England Biolabs) and subjected to quality control with an Agilent 2100 bioanalyser. The resulting samples were then submitted for single-cell RNA-seq on an Illumina HiSeq 2000 platform using the 100-bp paired-end sequencing strategy.

Out of 25 embryos chosen for sequencing, three embryos were excluded because of technical difficulties (no RNA recovered). We submitted a total of 22 embryos for sequencing, which were classified as viable or non-viable based on mechanical parameters.

### RNA-seq data processing

Sequencing returned ∼1 billion paired-end reads of length 101 belonging to 22 embryos total. Raw sequence data in fastq format were passed through quality control to remove low-quality base calls and adapter sequences (FastX trimmer and clipper). The filtered sequences were then aligned to the reference human genome (hg18) with the STAR aligner tool^67^ on a computer. The resulting .bam files were filtered for alignment quality, sorted and PCR duplicates removed (SAMtools sort and rmdup). Counts per gene were then calculated using HTSeq-count^68^ and comparing the aligned reads to the reference human transcriptome.

To account for possible batch effects arising from biological variation between patients, different freezing protocols, culture media or other factors, we had to determine which embryos came from which patients. We analysed sequence variants in the transcriptomes of each embryo, and performed hierarchical clustering on the variants for which we had good sequencing coverage (Supplementary Fig. 11). We determined that of the 22 embryos sequenced, 17 had ‘sibling' embryos, while 5 were the only embryos from their respective patients. We excluded those five, and then used the ComBat function in the sva^69^,^70^ package in R to remove batch effects from the RNA-seq data from the remaining 17 embryos. Although excluding the five embryos without siblings may have biased our results, it would have been impossible to estimate biological variation within those patients using only one sample and therefore we had to exclude them. After the removal of these batch effects, our data reflected differences in maternally inherited transcripts between viable and non-viable embryos consistent across all patients. Differential expression analysis was conducted on the adjusted data using the R package edgeR^28^. Genes with *q*-values (*P* values adjusted by the Benjamini–Hochberg method) less than 0.01 were classified as statistically significant.

### Gene ontology and network analysis

The list of DE genes with *q*-values<0.01 was passed into DAVID tool (NCBI), and clustering was performed with ontology terms related to BPs and molecular functions (MFs). Clusters with at least one term with an adjusted *P* value<0.05 were deemed statistically significant and included in Table 1. Terms within each cluster were combined into a single descriptive phrase and included in Table 1. Further gene ontology analysis was conducted with the goseq and GO.db packages in R. All gene ontology categories with at least 10 genes were ranked in order of the percentage of genes DE within that category, and compared with the proportion of genes DE across all categories.

IPA was used to analyse the list of DE genes along with the log-fold change between viable and non-viable embryos. A core analysis was conducted to find cellular processes that were predicted to be significantly increased or decreased based on gene expression changes as well as networks of genes that were regulated as a group.

### Cortical granule staining

The mechanical parameters of embryos from B6 mice and embryos from IVF (from CBA mice) were measured at the zygote stage in two separate experiments. The embryos were classified as viable or non-viable based on their mechanical parameters, and then ZP was removed by briefly exposing embryos to Tyrode's solution (Sigma) followed by three washes in PBS (Invitrogen). Denuded embryos were then fixed in 4% paraformaldehyde for 20 min at room temperature and stained with fluorescein isothiocyanate-labelled Lectin antibody (Sigma) and 4,6-diamidino-2-phenylindole. A Zeiss LSM510 laser-scanning confocal microscope was used for visualization and image capture.

### Confocal image processing

Raw image stacks were converted to TIFF format and custom Matlab scripts were written to extract image parameters. Contrast adjustment was carried out on all images in each stack to account for the loss of signal at greater imaging depths, and a projection image was calculated for each stack. The cytoplasm of each cell was isolated by performing automatic thresholding using Otsu's method, Canny edge detection and a circle Hough transform to detect the location and size of each cell. An intensity profile was calculated along a path around the circumference of each cell, at 95% of the cell radius, and with width of 10% of the cell radius. The parameters plotted in image 4 were (a) the average and (b) s.d. of this intensity profile. The investigator was blinded to each embryo's mechanical parameters and group (in the case of the microinjection experiments) while measuring cortical granule parameters.

### Statistical tests

A *χ*^2^-test was used to test whether the proportion of embryos resulting in live birth (section 2.2) was significantly different between the three experimental groups (predicted viable based on mechanics, predicted non-viable based on mechanics and control-sorted only by morphology). The only assumption made by this test is that no group (a `group' refers to either embryos resulting in live birth, or embryos not resulting in live birth) contains less than five samples. This assumption is met for both comparisons that were performed (viable versus control, viable versus non-viable).

To determine genes with significant differences in expression between viable and non-viable human embryos, the sva and edgeR packages in R were used. These packages have been well validated for use in removing batch effects and determining significant differences in expression for RNA-seq data.

A Wilcoxon rank sum test was used to compare cortical granule brightness between embryos (a *t*-test was not used because samples had low numbers and were not normally distributed according to the Lilliefors test). To determine whether injection of the IP3 antibody changed the mean embryo stiffness, a two-sided *t*-test was used because the antibody-injected and control-injected groups were determined to be normally distributed using the Lilliefors test. A two-sample Kolmogorov–Smirnov test was used to show that the injection of the antibody caused a significant change in the distribution of stiffness values. Wilcoxon rank sum tests were used to compare differences between quartiles of the antibody-injected and control-injected groups because there were very few samples in each quartile. Wilcoxon rank sum tests were also used to test the difference in the median values of oocyte stiffness between the GV, MI and MII stages because the stiffness values did not meet the criteria for normality.

## Additional information

**Accession codes**: The raw and processed RNA-seq data used in this study has been deposited in the Gene Expression Omnibus (GEO) database under accession code GSE65481. All R scripts used to process RNA-seq data and conduct differential expression analysis can be found in the GitHub repository https://github.com/liviaz/EmbryoProject in the RNA_seq_analysis folder.

**How to cite this article:** Yanez, L. *et al*. Human oocyte developmental potential is predicted by mechanical properties within hours after fertilization. *Nat. Commun.* 7:10809 doi: 10.1038/ncomms10809 (2016).

## Supplementary Material

Supplementary Information Supplementary Figures 1-11 and Supplementary Table 1

## Figures and Tables

**Figure 1:**
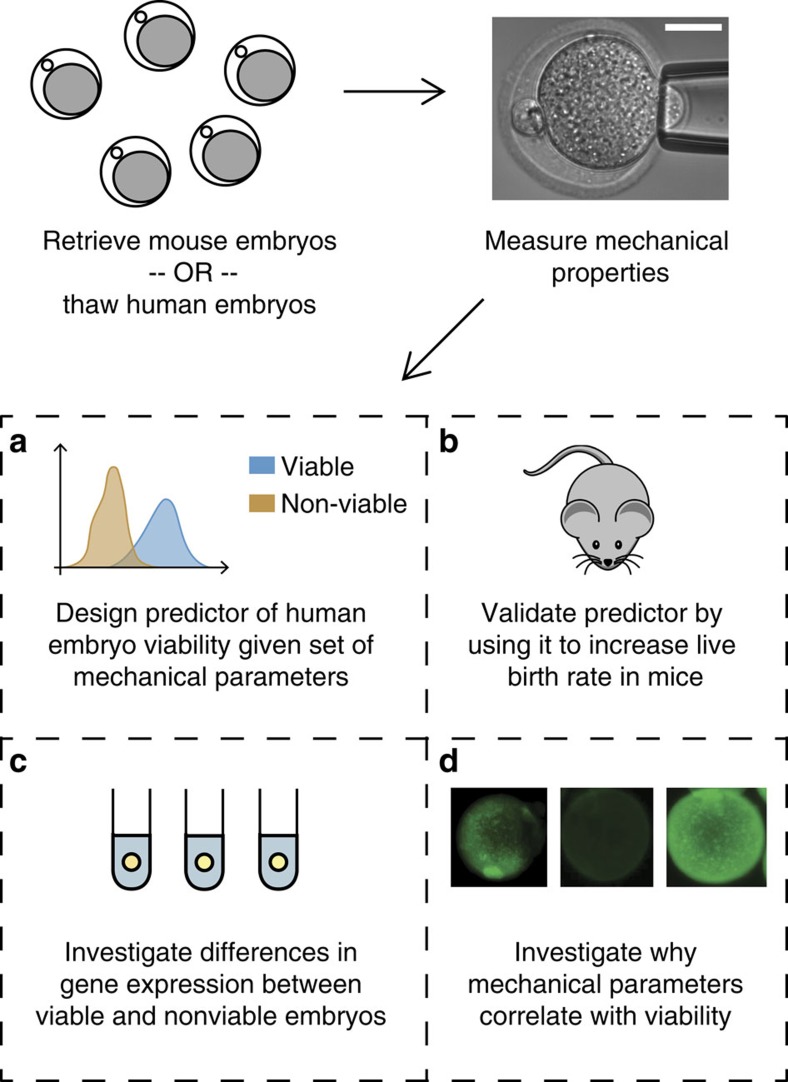
Overview of experimental design. (**a**) We measured mechanical properties of 282 mouse and 89 human zygotes, and found the combination most predictive of viability, defined as survival to the blastocyst stage. (**b**) We then measured live birth rates in mice that received either embryos that were predicted to be viable based on mechanics (*n*=55), predicted non-viable based on mechanics (*n*=55) or randomly chosen as a control (*n*=55). (**c**) We conducted single-cell RNA-seq on 17 human zygotes and found differences in transcriptomes between those predicted to be viable and non-viable based on mechanics. (**d**) We investigated differences in cortical granule release between 30 embryos predicted to be viable and 37 embryos predicted to be non-viable based on mechanics. Scale bar, 40 μm.

**Table 1:** Gene ontology analysis of differentially expressed genes.

	**Cluster name**	**Cluster enrichment**	**Min** ***q-*****value in cluster**	**Maturation involvement**
1	Nucleotide binding	9.32	4.0e−10	Cytoplasmic
2	DNA repair, response to DNA damage	6.37	1.2e−4	Not applicable
3	Protein catabolic process	5.73	1.1e −4	Cytoplasmic
4	Cell cycle and mitosis	5.71	4.5e −5	Nuclear
5	Metal ion binding	4.47	6.3e− 5	Cytoplasmic
6	mRNA processing and splicing	4.22	2.7e −4	Cytoplasmic
7	Regulation of transcription	3.9	5.6e −5	Cytoplasmic
8	Intracellular transport and protein localization	3.66	6.3e− 4	Cytoplasmic
9	Phosphatidylinositol signalling system	3.49	8.0e −4	Cytoplasmic
10	ATPase and DNA helicase activity	2.93	9.4e −3	Nuclear
11	Protein modification and ubiquitination	2.85	1.8e −3	Cytoplasmic
12	Chromatin modification and chromosome organization	2.75	1.9e −2	Cytoplasmic
13	Protein serine/threonine kinase activity	2.64	1.4e −2	Cytoplasmic
14	Microtubule-based movement	2.6	2.9e −2	Cytoplasmic
15	Coenzyme metabolic process	2.58	1.9e− 2	Cytoplasmic
16	Protein complex assembly	2.24	8.2e −3	Cytoplasmic
17	Sister chromatid segregation and spindle formation	2.2	4.7e −2	Nuclear
18	Transcription initiation and transcription factor activity	1.79	2.3e− 4	Cytoplasmic
19	Proteasomal protein catabolic process	1.05	4.7e −2	Cytoplasmic
