# Supplementary material for: Human oocyte developmental potential is predicted by mechanical properties within hours after fertilization
Source: Nat Commun. 2016 Feb 24;7:10809. doi: 10.1038/ncomms10809 (PMC4770082; doi:10.1038/ncomms10809)
Supplement: Supplementary Information — Supplementary Figures 1-11 and Supplementary Table 1 [file ncomms10809-s1.pdf]

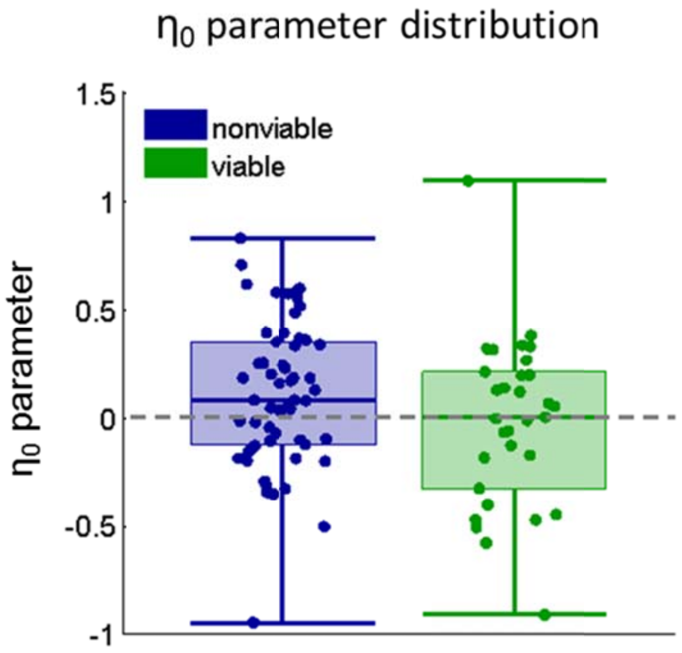

Supplementary Figure 1: Comparison of η_0 parameter between viable and nonviable human zygotes. Unlike with the other parameters, viable zygotes (n=31) did not have a narrower distribution than nonviable zygotes (n=58) (p = ns, Wilcoxon rank sum test). Error bars represent standard deviation.

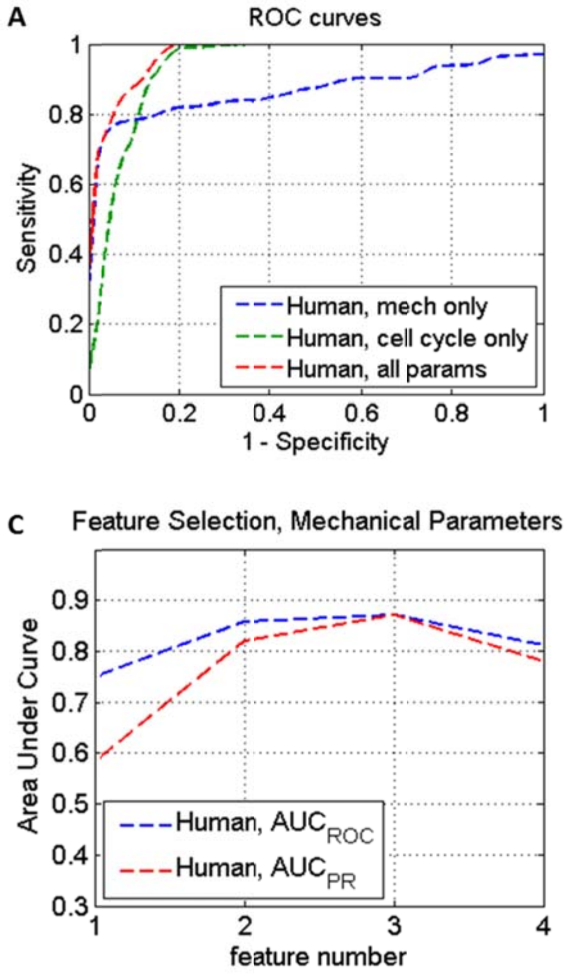

Supplementary Figure 2: Evaluating classification performance when predicting human embryo viability. A) ROC curves for human embryo mechanical parameters alone, cell cycle parameters alone, and the combination of all parameters. B) Precision-recall curves for human embryo mechanical parameters alone, cell cycle parameters alone, and the combination of all parameters. C) Feature selection for human mechanical parameters showing that 3 parameters (k_1, n_1, k_0) offer the optimal predictive value. D) Human embryo mechanical parameters provide good predictive value on their own, which is improved by adding cell cycle parameters as they become available over time.

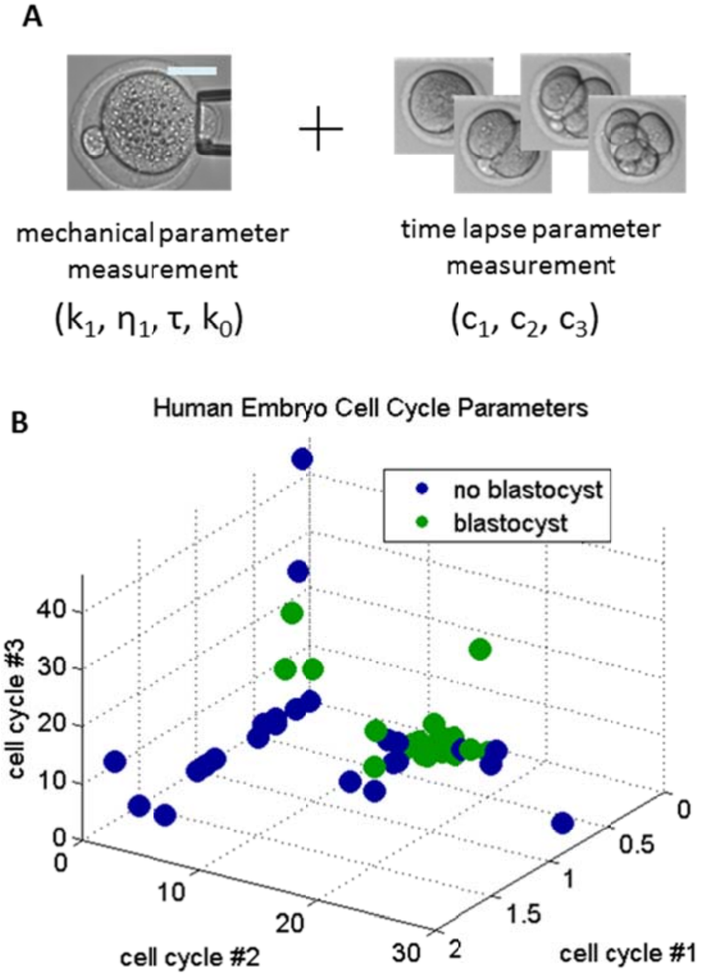

Supplementary Figure 3: Combination of mechanical and cell cycle parameters. A) We compared the effectiveness of mechanical parameters and cell cycle parameters for predicting viability. Scale bar = 40 µm. B) Cell cycle parameters for human embryos in our dataset (n=89).

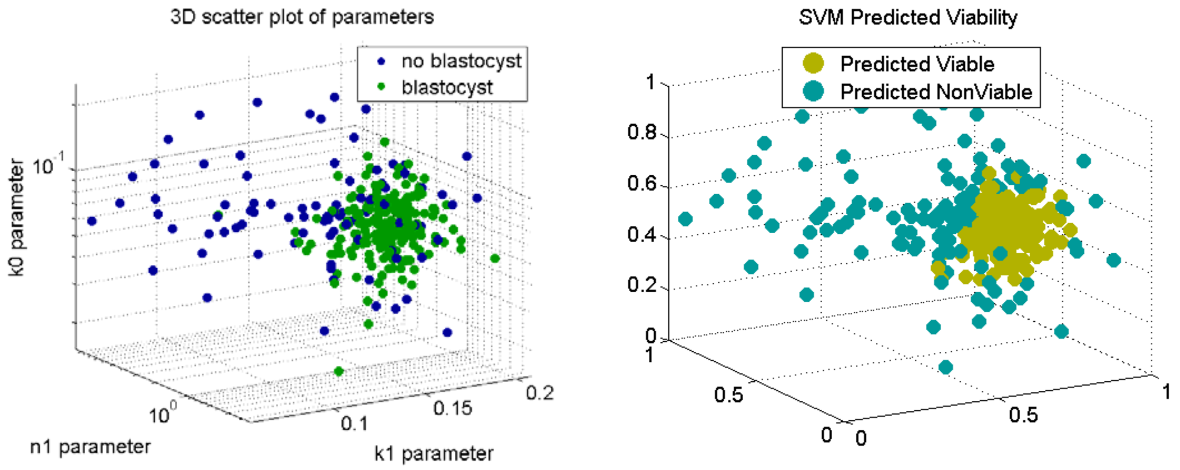

Supplementary Figure 4: Example of embryo viability classification based on mechanical parameters. A 3D plot of mouse zygote mechanical parameters (n=282) is shown on the left side, along with an example viable/nonviable classification using support vector machines (SVM) on the right side.

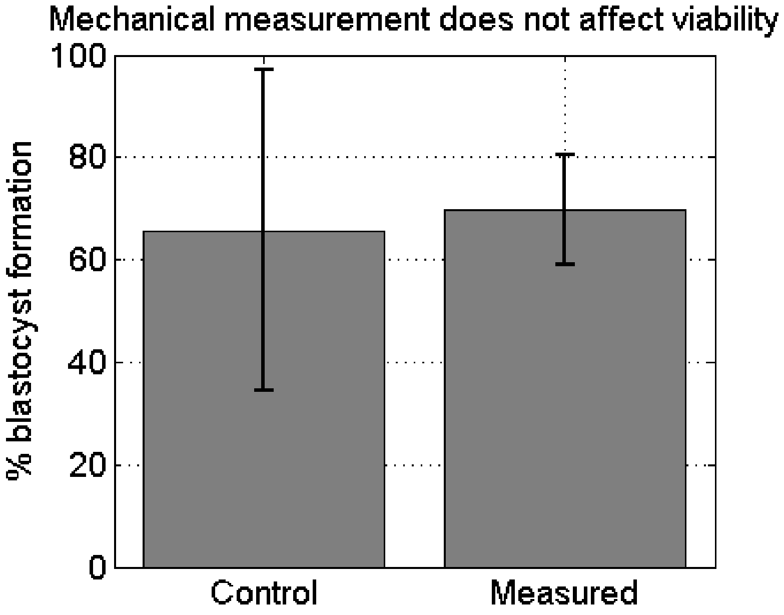

Supplementary Figure 5: Comparison of blastocyst formation rates between control and measured embryos. Blastocyst formation rates are not significantly different (p = 0.62 using two-proportion Z-test) between control (n = 35) and measured (n = 282) mouse 2PN embryos. Error bars represent 95% CI.

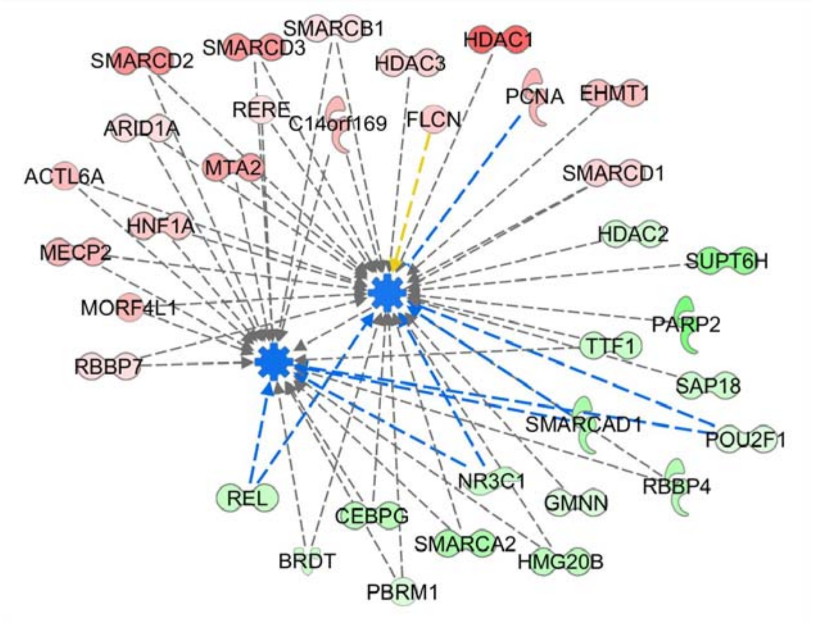

Supplementary Figure 6: Result of IPA analysis predicting that nonviable embryos have decreased levels of chromatin remodeling and regulation of chromosome organization.

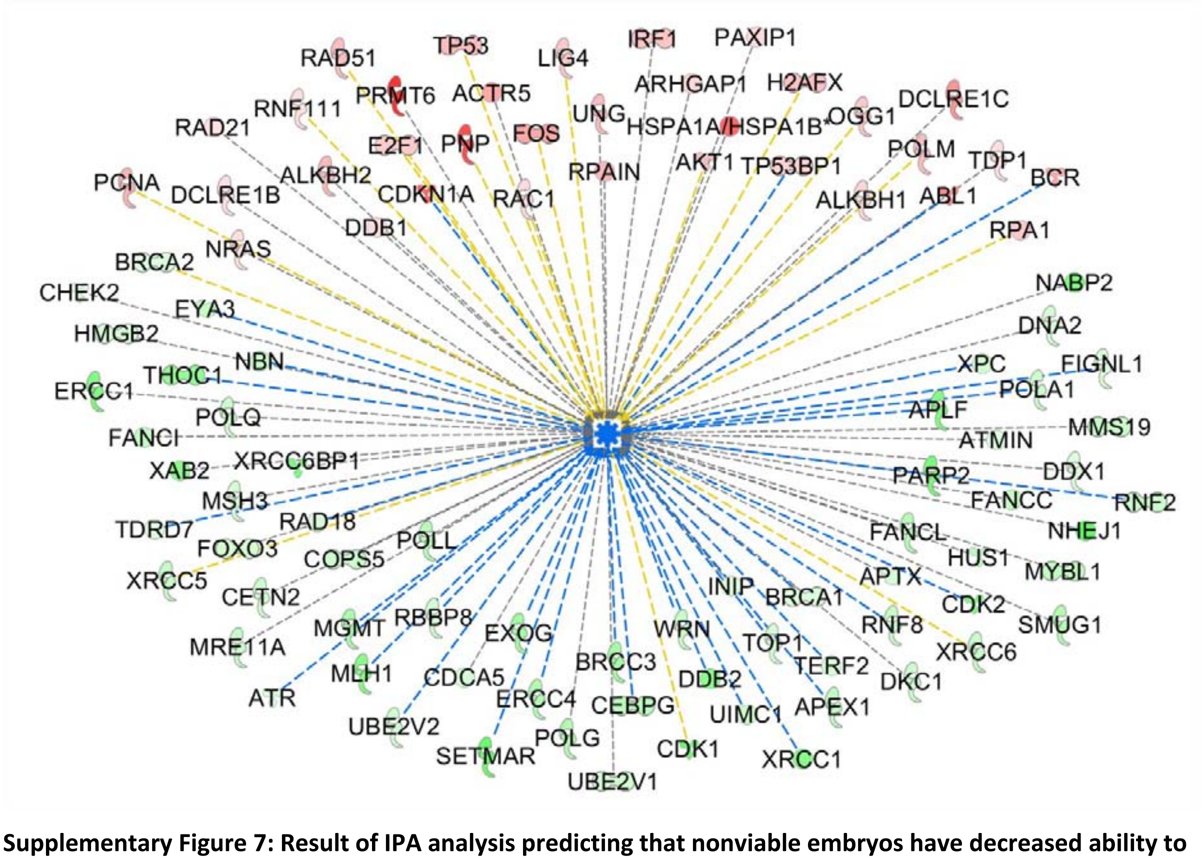

Supplementary Figure 7: Result of IPA analysis predicting that nonviable embryos have decreased ability to repair DNA damage.

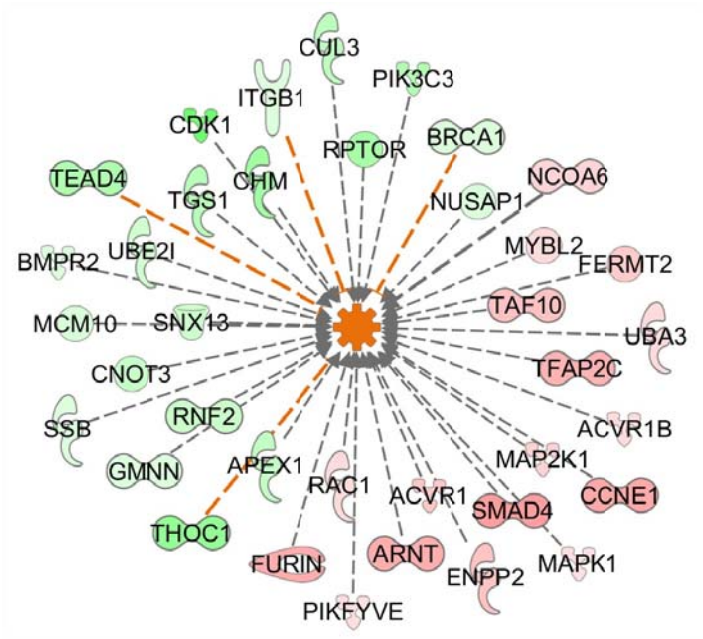

Supplementary Figure 8: Result of IPA analysis showing that nonviable embryos are more likely to have poor blastocyst morphology

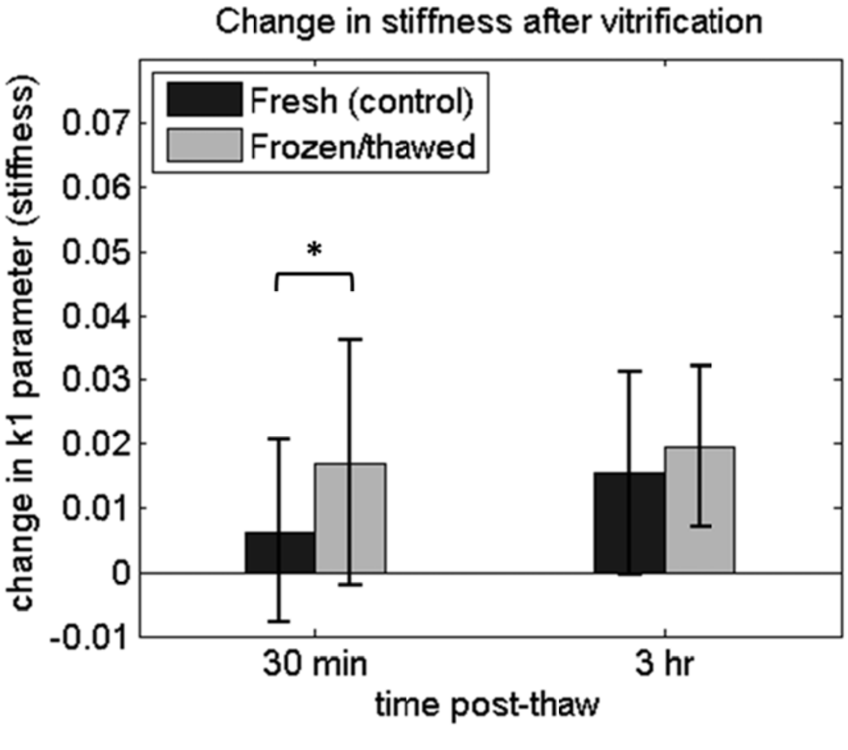

Supplementary Figure 9: Comparison of vitrification-induced changes in mouse embryo stiffness. Mouse embryos exhibit increased stiffness directly after freezing and thawing (fresh: n=20; frozen/thawed: n=22, $p < 0.05$, Wilcoxon rank sum test), but stiffness values return to normal by 3 hrs post-thaw (fresh: n=20; frozen/thawed: n=13, p = ns, Wilcoxon rank sum test). The y-axis represents the change in the k1 parameter relative to a pre-thaw measurement.

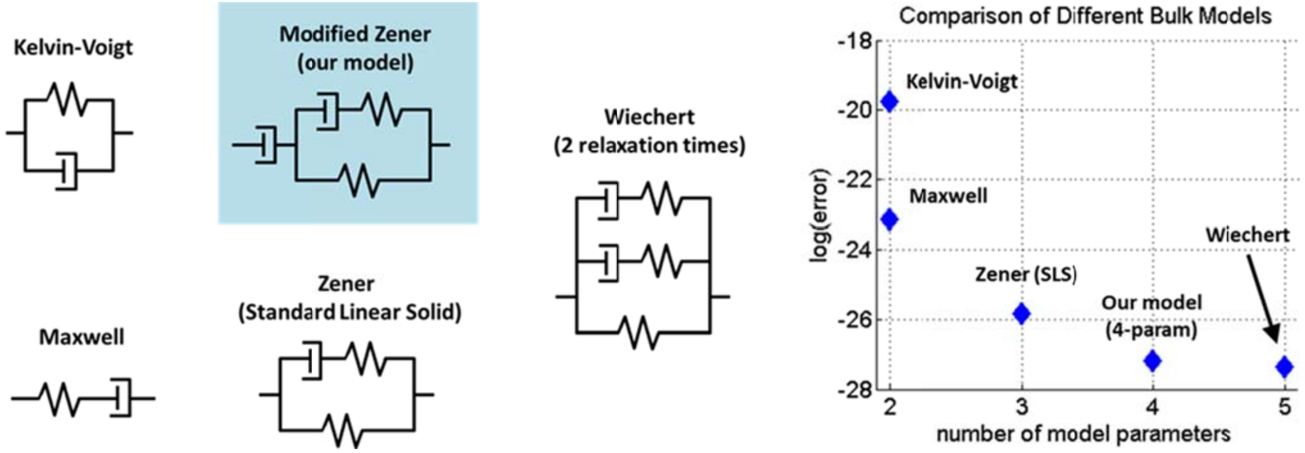

Supplementary Figure 10: Comparison of fitting error for 5 different bulk mechanical models. Adding more parameters to the model we chose does not significantly lower the fitting error, so our model is able to achieve good performance without overfitting.

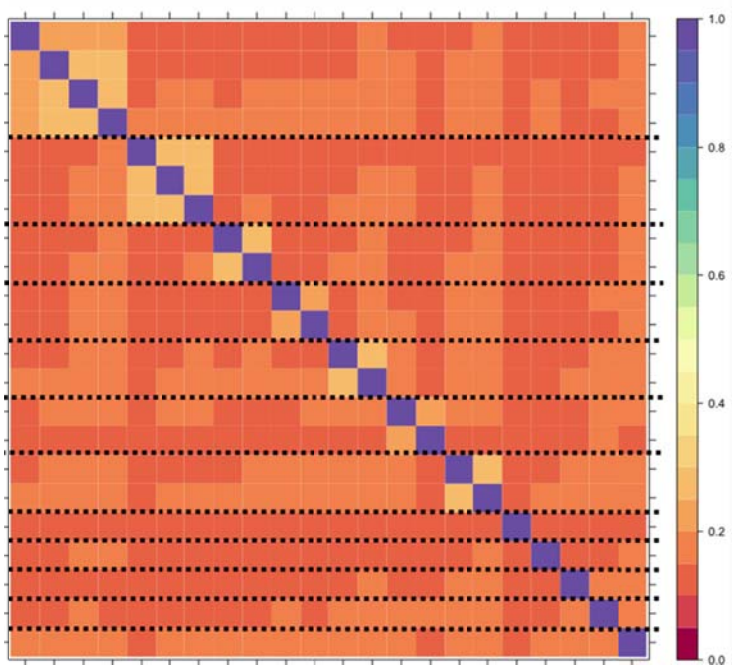

Supplementary Figure 11: Hierarchical clustering of SNVs from RNA-seq data. The horizontal black lines show how sibling embryos were grouped. The 5 embryos at the bottom had no siblings and were thus excluded from analysis.

Trial	# Embryos Transferred to Each Mouse	% of viable embryos resulting in live birth (proportion)	% of nonviable embryos resulting in live birth (proportion)	% of control embryos resulting in live birth (proportion)
1	14	64% (9/14)	21% (3/14)	43% (6/14)
2	12	67% (8/12)	17% (2/12)	50% (6/12)
3	14	71% (10/14)	14% (2/14)	50% (7/14)
4	15	80% (12/15)	40% (6/15)	53% (8/15)
Total	55	71% (39/55)	24% (13/55)	49% (27/55)

Supplementary Table 1: Mouse live birth experiments
